# Low-Protein Diet in Elderly Patients with Chronic Kidney Disease Stage 4 and 5 in Conservative Management: Focus on Sarcopenia Development

**DOI:** 10.3390/nu16101498

**Published:** 2024-05-16

**Authors:** Francesca K. Martino, Alessandra Zattarin, Chiara Cinquini, Silvia Toniazzo, Francesco Francini Pesenti, Lucia Federica Stefanelli, Martina Cacciapuoti, Elisabetta Bettin, Lorenzo A. Calò, Paolo Spinella

**Affiliations:** 1 Nephrology, Dialysis and Transplantation Unit, Department of Medicine (DIMED), University of Padova, 35128 Padua, Italy; luciafederica.stefanelli@unipd.it (L.F.S.); martina.cacciapuoti@unipd.it (M.C.); elisabetta.bettin@aopd.veneto.it (E.B.); 2 Department of Medicine (DIMED), Clinical Nutrition, University of Padua, 35128 Padua, Italy; alessandra.zattarin@aopd.veneto.it (A.Z.); chiara.cinquini@studenti.unipd.it (C.C.); silvia.toniazzo@aopd.veneto.it (S.T.); francesco.francini@aopd.veneto.it (F.F.P.); paolo.spinella@unipd.it (P.S.)

**Keywords:** chronic kidney disease, dietary interventions, low protein diet, sarcopenia

## Abstract

Introduction: Chronic kidney disease is a degenerative and increasingly prevalent condition that includes metabolic abnormalities and is associated with a higher risk of sarcopenia. The conservative approach points primarily to controlling metabolic issues and reducing the risk of malnutrition and sarcopenia, slowing the progression of kidney disease. The present study aims to evaluate the effect of a low-protein diet on malnutrition and sarcopenia. Methods: A total of 45 patients (33 male and 12 female) aged over 70 with chronic kidney disease stage 4–5 in conservative management were considered. All patients had a dietary assessment and prescription of personalized low-protein dietary plans (≤0.6 g protein/kg) and a follow-up control between 4 and 6 months. In preliminary and follow-up evaluations, anthropometric data, blood examinations, body composition results, muscle strength, physical performance, and a 3-day food diary were collected. Results: In the follow-up period, a significant weight loss (*p* = 0.001) and a decrease in body mass index (*p* = 0.002) were recorded. Food diaries revealed a significant reduction in protein, sodium, potassium, and phosphorus intake (*p* < 0.001), with a significant reduction in urea (*p* < 0.001) and proteinuria (*p* = 0.01) without any impact on lean mass (*p* = 0.66). Considerable variations in adherence between food diaries and the prescribed diet were also noted. Conclusions: Providing a personalized low-protein diet led to significant benefits in a short period without worsening the patient’s nutritional status.

## 1. Introduction

Chronic kidney disease is a globally increasing public health concern associated with aging and a higher prevalence of comorbidities, such as diabetes and hypertension [1]. Recent data from the International Society of Nephrology [2] indicate a global chronic kidney disease prevalence of 10%, even higher than diabetes and cancer rates. Especially in the last twenty years, there has been an increasing number of elderly patients who reach chronic kidney disease stages 4 and 5 and who are currently a prevalent group among chronic kidney disease patients in Western countries. The United States Renal Data System 2020 Annual Data Report showed, in the 2000 to 2020 period, a significant increase in patients over 75 years with chronic kidney disease stage 5 who need renal replacement therapy [3]. This phenomenon seems related to different conditions, such as the aging of the general population, which is associated with improving welfare and medical care, and the increase in diabetes, hypertension, and chronic vascular disease. In a recent report from the northeast district of Italy, about 2310 patients per million of the population reached chronic kidney disease stage 5, of whom the prevalent group is elderly patients [4]. In the same study, about 20% of the patients did not receive any nephrological care, likely due to issues with basal conditions. The optimization of nephrology care with the support of nutritional care could alleviate basal conditions in elderly patients who reach end-stage kidney disease, improving the quality of life and avoiding/delaying the need for renal replacement therapy.

Sarcopenia is a degenerative and progressive disorder of skeletal muscle characterized by a reduction in muscle mass and function, and it is associated with increased morbidity and mortality [5]. Aging, low body mass index, malnutrition, and an inflammatory state are risk factors for the development of sarcopenia [6]. Prevention and treatment strategies through aerobic and resistance exercises and personalized nutritional interventions are crucial to reduce sarcopenia risk [7].

The prevalence of sarcopenia in chronic kidney disease patients ranges from 4% to 42% and depends on definitions, studied populations, and disease stages [6,7,8]. The presence of chronic kidney disease is an independent risk factor for the development of sarcopenia [6,7,8]. Furthermore, conditions strongly related to chronic kidney disease, such as inflammation, metabolic acidosis, insulin resistance, and vitamin D deficiency, increase the sarcopenia risk [7,8,9]. Finally, some chronic kidney disease symptoms, such as the lack of appetite and taste impairment, could lead to malnutrition and consequentially to sarcopenia, especially in the late phase of chronic kidney disease.

A low-protein diet is a dietary therapy for chronic kidney disease characterized by a low content of proteins, sodium, phosphorus, and potassium [8,9,10]. The restrictions should be gradually applied according to the stage of the disease to correct protein and energy intake and, consequentially, to ensure its success and safety. Furthermore, the personalized diet should consider the presence of comorbidities and the patient’s needs. In the latest KDOQI guidelines for nutrition in CKD [10], it is specified that reducing protein intake can compromise the nutritional status in subjects at risk of protein-energy wasting, a clinical condition characterized by a decrease in body protein and energy reserves (involving loss of both lean and fat mass) and often associated with reduced functional capacity related to metabolic events [10]. However, it is also reported that, with sufficient energy intake (25–35 kcal/kg per day), the protein intake level can be reduced to 0.55–0.6 g of protein/kg per day without malnutrition. Therefore, high caloric intake is mandatory to prevent proteins from being used for energy and to prevent lean mass loss. In selected patients, further reduction in protein intake to 0.3–0.4 g of protein/kg per day is possible, providing they are supplemented with keto-analogues to ensure an adequate intake of essential amino acids.

Regarding mineral salt requirements, KDOQI does not provide specific references but suggests providing potassium and phosphorus intake to maintain normal blood levels. More precise and valuable references in clinical practice can be found in Italian documents for managing patients with CKD [11], where a phosphorus intake of 700 mg/day is suggested. KDOQI recommends keeping sodium intake below 100 mmol/day, equivalent to 2.3 g/day, a quantity also found in Italian documents.

A low-protein diet has demonstrated significant benefits in managing chronic kidney disease [12], slowing its progression and reducing symptoms [13,14]. Studies highlight that reduced protein intake is associated with metabolic improvements, such as increased bicarbonate and reduced phosphorus, and a lower risk of progressing to end-stage renal disease [13].

While a low-protein diet has shown promise in managing chronic kidney disease, it also carries potential risks. It could be a risk factor for malnutrition and sarcopenia, posing a disadvantage for chronic kidney disease patients. Striking the right balance between risks and benefits is crucial. In frail elderly patients with chronic kidney disease stages 4 and 5, a personalized low-protein diet could be a viable solution to manage chronic kidney disease and prevent protein-energy wasting and sarcopenia. However, careful consideration and monitoring are essential to mitigate the potential risks.

In this context, studies on the relationship between sarcopenia and a low-protein diet in chronic kidney disease patients are scant [9]. The present study aims to evaluate the role of a dietary approach with a low-protein diet in elderly patients with chronic kidney disease stages 4 and 5, focusing on metabolic chronic kidney disease control and the risk of sarcopenia over four months.

## 2. Materials and Methods

We conducted a retrospective observational study on a cohort of chronic kidney disease patients over 70 undergoing conservative management at the Nephrology Unit of Padua University Hospital between 1 January 2023 and 1 April 2023. All patients with a preliminary assessment and a subsequent dietary evaluation after four to six months were considered adequate for the enrolment. The exclusion criterion was a lack of informed consent to participate in the study. Notably, during the enrolment period, 58 patients had their first dietician evaluation; among the 13 patients who were lost to follow-up, 2 started replacement therapy, 8 were hospitalized during the follow-up visit period, and 3 discontinued the program for personal reasons.

Written informed consent was collected for each patient, and a unique code was used to analyze the data anonymously and ensure patient privacy. The study was conducted according to the Declaration of Helsinki and with the approval of the ethics committee of Padua University Hospital (Code CET:426n/AO/23).

The following features were evaluated in each patient:

- Epidemiological parameter evaluations included age, gender, and medical comorbidities such as diabetes, hypertension, dyslipidemia).
- The anthropometric data included weight, height, and body mass index.
- Bioelectrical impedance analysis (Akern^®^ BIA 101 New Edition-sinusoidal 50 kHz waveform current, intensity 0.8 A) was used to analyze body composition, particularly appendicular skeletal muscle mass. Data were analyzed through the software Bodygram^®^ Dashboard by Akern Srl V. 1.0.
- Muscle strength was measured through the Handgrip test (Jamar Hydraulic Hand Dynamometer—5030J1 by Petterson Medical; dual-scale readout displays isometric grip force from 0 to 200 lbs [90 kg]).
- Physical performance was evaluated through the Gait Speed test (measurement of walking speed by timing the time it takes to cover a distance of 4 m).

In cooperation with the patient, we reviewed a dietary diary completed at home three days before the visit. The data on nutrient intakes (calories, proteins, carbohydrates, lipids, fibers, sodium, phosphorus, and potassium) were obtained using the Italian food composition tables [15]. The intakes were calculated using the Metadieta software (Meteda-METEDA S.r.l. Via S. Pellico, 4 63074 San Benedetto del Tronto (AP), Italy, https://www.metadieta.it/, accessed on 1 March 2024).

Biochemical parameters included creatinine, plasma urea, uric acid, serum sodium, potassium, calcium, phosphorus, albumin, hemoglobin, transferrin saturation, blood glucose, and proteinuria. These parameters were measured about two weeks before the respective visits.

### 2.1. Dietary Intervention

Each patient had a personalized low-protein diet, according to the KDOQI suggestions. Specifically, the main characteristics of energy, macro- and micronutrient content, and whether supplementation was needed were as follows:

- Energy: 25–30 kcal/kg/day;
- Proteins: 0.3–0.7 g/kg/day. A very low-protein diet (0.3–0.4 g/kg/day) was supported by essential amino acids or keto-analogues, according to patient weight;
- Carbohydrates: At least 60% of total energy intake;
- Lipids: At least 30% of total energy intake;
- Sodium: 2.3 g/day (corresponding to 6 g of NaCl);
- Phosphorus and potassium: adequate to maintain normal blood levels;
- Supplementations: carbonate calcium, vitamins, iron.

### 2.2. Sarcopenia Definition

According to the European Working Group on Sarcopenia in Older People 2 [3], the diagnosis of sarcopenia is based on low muscle strength (handgrip < 27 kg for men and <16 kg for women) and a low muscle mass (appendicular skeletal muscle mass < 7 kg/m^2^ for men and <5.5 kg/m^2^ for women). The presence of reduced physical performance (gait speed < 0.8 m/s) is an index of severe sarcopenia.

### 2.3. Statistical Analysis

According to their distribution, numeric variables were reported using either the mean and standard deviation or the median and standard deviation. Categorical variables were presented as percentages and absolute numbers. Comparisons between measurements at T0 and T1 were assessed using paired t-tests for normally distributed variables or the Wilcoxon signed-rank test for non-normally distributed variables. Depending on their distribution, correlations among numerical variables were evaluated using Pearson or Spearman correlation coefficients.

Differences were considered significant at *p* < 0.05.

The analyses were conducted using SPSS software version 28.

### 2.4. Sample Size

Considering the available studies and their characteristics and the lack of studies that compare body muscle mass before and after the beginning of a low-protein diet, we estimated the sample size according to the report about muscle function in stage 3–5 chronic kidney disease patients with a low-protein diet [16]. We used the results about muscle mass, which were 29.68 ± 5.06% in the low-protein diet group and 34.82 ± 8.29% in the no-protein restriction diet group.

The sample size of the study was estimated using the OpenEpi calculator (https://www.openepi.com/SampleSize/SSCohort.htm, accessed on 1 December 2022), comparing the means of muscle mass with a power of 80% and confidence interval (2-sided) of 95%.

The sample size formulae used were as follows:

n_1_ = (σ_1_^2^ + σ_2_^2^/k) (Z_1−α/2_ + Z_1−β_)^2^/Δ^2^

n_2_ = (k * σ_1_^2^ + σ_2_^2^) (Z_1−α/2_ + Z_1−β_)^2^/Δ^2^

where n_1_ = sample size of Group with a low-protein diet; n_2_ = sample size of Group without a low-protein diet; σ_1_ = standard deviation of Group 1; σ_2_ = standard deviation of Group; Δ = difference in group means; k = ratio = n_2_/n_1_, Z_1−α/2_ = two-sided Z value; Z_1−β_ = power.

We obtained a sample size of 29 patients for each group.

Considering the characteristics of our study, where each subject represented the control before the beginning of a low-protein diet and the case after 4–6 months of a low-protein diet, we assumed n1 was equal to n_2_.

Therefore, to avoid selection bias in our cohort, all patients who satisfied the inclusion and exclusion criteria in a three-month enrollment period between 1 January 2023 and 1 April 2023 were enrolled.

## 3. Results

A total of 45 patients (33 male and 12 female) with an average age of 78 years (±6.2) were included in the study. The examined patients had an average age of 78 years (±6.2). Among the associated comorbidities, 40% of the sample was found to have diabetes (18 patients), 51.1% had hypertension (23 patients), and 13.3% had dyslipidemia (6 patients). Patients without any comorbidities accounted for 26.7% (12 patients), those with only one comorbidity accounted for 44.4% (20 patients), those with two comorbidities accounted for 22.2% (10 patients), while 4.4% (2 patients) presented all three comorbidities. Table 1 reports the main characteristics of our cohort.

In the follow-up period, patients showed a significant weight loss (74.5 ± 15.9 kg vs. 72.9 ± 15.5 kg, *p* = 0.001) with an expected significant decrease in body mass index (28 ± 5.7 kg/m^2^ vs. 27.4 ± 5.6 kg/m^2^, *p* = 0.002) and a substantial reduction in fat mass (19.5 [13.9–29.6] vs. 18 [10.9–27.7], *p* < 0.001). Body composition assessment through BIA revealed a non-significant change between basal and follow-up observation in lean mass (52.3 ± 10.8 kg vs. 53 ± 8.5 kg), in extracellular water (24.6 ± 7.7 L vs. 23.2 ± 3.9 L), total body water (41.5 ± 7.6 L vs. 41.4 ± 7.1 L), and phase angle (4.3 ± 0.7° vs. 4.2 ± 0.7°), while we found a significant decrease in fat mass (21.3 ± 9.8 kg vs. 19.8 ± 9.5 kg, *p* < 0.001). Table 2 reported anthropometric and body composition data.

Out of the 45 examined patients, 6 (all male) were classified as sarcopenic according to the European Working Group on Sarcopenia in Older People 2 [3] criteria, with a prevalence of 13.3% both at baseline and after four months, as reported in Table 3. One of these six was classified as severely sarcopenic. Table 3 reports all sarcopenia criteria at the beginning and the end of the observational period. Sarcopenia was found to be associated with weight and body mass index, showing a significant negative correlation (rho *−*0.33 *p* = 0.03 and rho *−*0.41 *p* = 0.047, respectively), while age and phase angle showed no significant correlation (rho 0.2 *p* = 0.64 and rho *−*0.18 *p* = 0.36, respectively).

In the follow-up period, the patients showed a significant decrease in plasma urea (22.5 ± 7 mmol/l vs. 17.9 ± 5.9 mmol/l, *p* < 0.001) and 24 h urine protein (1.02 [0.53–2.85] vs. 0.57 [0.29–2.14], *p* = 0.01). However, no significant differences emerged between the values at T0 and T1 for serum phosphate, calcium, albumin, and hemoglobin. Table 4 reports blood examination results in the follow-up period.

In our cohort, 91.1% of patients (42 individuals) used protein-free products. Protein, sodium, potassium, and phosphorus intake significantly decreased. Conversely, lipid, carbohydrate, and fiber intake increased significantly. Table 5 presents the assessment of dietary habits through a 3-day food diary.

**Figure 1:**
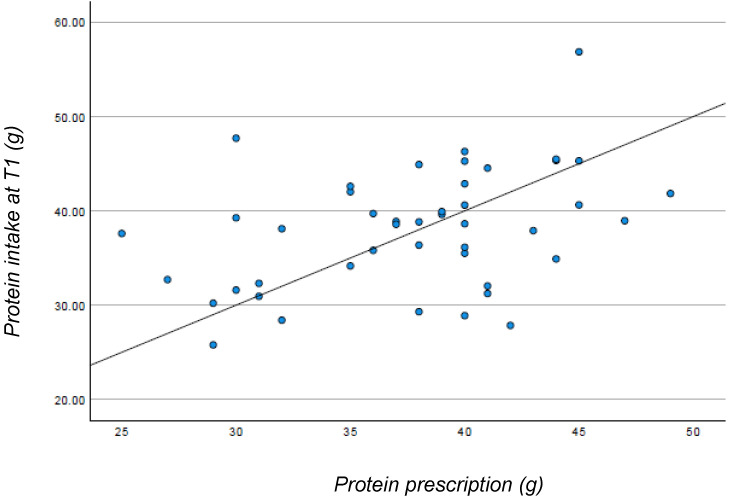
Relationship between protein prescription and protein intake at T1.

Adherence to the diet was evaluated through correlations between the prescribed protein content and the protein content reported in the three-day food diary. After four months of follow-up, the mean protein intake was 38 ± 6.3 g, in line with the average prescribed protein of 38 ± 5.7 g. The prescribed protein and the actual protein intake according to the food diary showed a significant correlation, with a Spearman rho equal to 0.367 (*p* = 0.013). However, the scatterplot (Figure 1) depicts a dispersion of the data, indicating variations among patients in their adherence to the prescribed diet.

## 4. Discussion

Our study showed that a low-protein diet had no short-term impact on sarcopenia in chronic kidney disease patients but impacted positively on the control of urea and lower urinary protein excretion.

Regarding the nutritional status of patients, our data show no significant worsening in body composition or biochemical parameters. Despite a significant weight loss and a subsequent decrease in body mass index observed across the entire sample, a detailed examination reveals that weight loss is significant only in overweight and obese patients with a body mass index > 25 kg/m^2^ (average loss of 2.2 ± 3.8 kg). Patients with a body mass index < 25 kg/m^2^ remained nearly stable (average loss of 0.5 ± 2 kg), indicating a non-significant decrease. Body mass index also showed a significant decrease only in subjects with a body mass index > 25 kg/m^2^. Weight loss is reported as a possible adverse outcome of a low-protein diet, especially in the first period, generally followed by weight stabilization [17,18]. Although comprehensive studies on weight loss are still limited, some reports agree that a low-protein diet, accompanied by initial weight loss, does not increase the long-term risk of protein-energy wasting [18,19]. Bioimpedance analysis showed a significant reduction in fat mass but not in lean mass or appendicular skeletal muscle mass. This observation suggests patients did not experience excessive muscle mass loss despite their age and low-protein diet. Additionally, parameters recommended by the European Working Group on Sarcopenia in Older People 2 [5] for sarcopenia diagnosis (handgrip, appendicular skeletal muscle mass, and gait speed) remained relatively stable. Our results are congruent with a previous study that reported that a low protein intake, when well-planned within personalized dietary therapy, can lead to several positive metabolic adaptations in chronic kidney disease patients, improving muscle protein turnover [20].

Finally, it is noteworthy that approximately 13% of patients had sarcopenia before starting the diet. This finding aligns with a recent review on sarcopenia in chronic kidney disease, reporting a prevalence of sarcopenia in non-dialysis chronic kidney disease patients between 5.9% and 14% (according to the European Working Group on Sarcopenia in Older People 2 criteria) [8].

Furthermore, weight and body mass index showed a significant negative correlation with sarcopenia, corroborating the previous report on the higher risk of developing sarcopenia in patients with lower weight and body mass index [6]. In this scenario, closer follow-up in patients with a lower body mass index seems mandatory to avoid sarcopenia and protein-energy wasting.

Analysis of estimated dietary intake through a 3-day food diary revealed a significant decrease in protein, sodium, phosphorus, and potassium intake, even with an equivalent energy intake. Specifically, protein intake decreased by approximately 40%. There was a significant increase in the intake of other macronutrients (carbohydrates, lipids, fibers), indicating a change in the patient’s diet composition. This change is justified by the high prevalence (91% of patients) of using protein-free products, which are rich in carbohydrates, mainly composed of starch, and often supplemented with fibers. An energy intake of about 25 kcal/kg body weight was observed. This value is at the lower limit of the recommended 25–35 kcal energy intake according to guidelines [8] and could explain the observed trend toward weight loss. The lower energy intake has been noted in other studies, as low as 22 kcal/kg body weight [21]. This highlights the difficulty for these individuals in maintaining an adequate energy intake, a challenge also reported during the visits with the dietitian, where many patients mentioned difficulty consuming all the prescribed foods.

Adherence to protein intake was higher, with a mean intake of 0.57 g/kg body weight. However, despite a linear relationship between prescribed and consumed proteins, there is a wide dispersion between expected and prescribed intake, indicating different patient adherence levels. Therefore, the 3-day food diary can be a valuable tool in determining which patients may need more support in adhering to the diet.

Finally, our results suggested a significant decrease in plasma urea levels, confirming previous studies about low-protein diets [22]. The 29.7% reduction in proteinuria is consistent with previous findings [21,23]. Initial improvements were seen as early as one week into a low-protein diet, with the best results (20–50% reduction in proteinuria) reported around three months of nutritional treatment.

The present study had some limitations. Firstly, the retrospective nature of the study represents a lower level of evidence. However, the close collaboration between nephrologists and dieticians in a new outpatient clinic allowed us to collect extremely detailed data about nephrological and nutritional features, with few missing data. Secondly, 13 patients were lost during follow-up for different reasons, which could be partially related to difficulties in managing a low-protein diet. Therefore, definitive conclusions regarding real adherence to the diet cannot be provided. Especially for the elderly, diet management could be complex, so evaluating patients’ difficulties in the future seems an important step to assess the feasibility of this class of patients. Thirdly, we used bioelectrical impedance analysis, which can slightly underestimate fat mass and overestimate free fat mass in obese people compared to other tools, such as dual-energy X-ray absorptiometry. In any case, even in obese people, bioelectrical impedance analysis in a supine position seems an adequate tool to assess body composition change, with excellent agreement with gold-standard methods [24].

## 5. Conclusions

The results of this study highlight the importance of implementing a low-protein dietary therapy in the conservative management of chronic kidney disease. A low-protein diet effectively improved nitrogen retention levels and reduced proteinuria in chronic kidney disease stages 4 and 5 without deteriorating nutritional status. More outstanding efforts should be directed toward optimizing adherence to the prescribed dietary treatment, especially concerning energy intake, which, particularly in the case of a low-protein diet, must be adequate to prevent lean mass loss and deterioration of overall long-term health.

As a future perspective, we plan to better understand the impact of a low-protein diet in the long term (12–24 months) with further analysis of our population.

## Figures and Tables

**Table 1:** Basal characteristics of population.

	n.(%)
Male	33 (73.3%)
Age (years)	78 (±6.2)
**Comorbidities**	
Diabetes	18 (40%)
Hypertension	23 (51.1%)
Dyslipidemia	6 (13.3%)

n. number of patients. Variables reported as absolute numbers and percentages n.(%).

**Table 2:** Anthropometric data and body composition at T0 and T1.

	n.	T0	T1	*p*-Value
**Weight (kg)** ^(1)^	45	74.5 (±15.9)	72.9 (±15.5)	**0.002**
- Weight with BMI < 25 kg/m^2^ (kg)	15	58.4 (±9.5)	57.9 (±8.9)	0.37
- Weight with BMI > 25 kg/m^2^ (kg)	30	82.6 (±11.8)	80.4 (±12.4)	**0.003**
**BMI (kg/m^2^)** ^(1)^	45	28 (±5.7)	27.4 (±5.6)	**0.003**
- BMI < 25 (kg/m^2^)	15	22 (±2.3)	21.8 (±2.1)	0.47
- BMI > 25 (kg/m^2^)	30	31 (±4.3)	30.2 (±4.5)	**0.004**
**BIA**				
- Reactance (Ohm) ^(2)^	45	454 [414–519]	452 [416–502.2]	0.12
- Resistance (Ohm) ^(2)^	45	35.1 [29.2–39.8]	34.2 [29.2–38.4]	0.16
- Phase Angle (°) ^(2)^	45	4.2 [3.7–4.8]	4.2 [3.7–4.7]	0.46
Lean Mass (kg) ^(2)^	45	54 [46.1–59.8]	54.1 [47.2–58.7]	0.66
Fat mass (kg) ^(2)^	45	19.5 [13.9–29.6]	18 [10.9–27.7]	**<0.001**
Total Body Water (L) ^(2)^	45	41 [37.2–47.5]	40.9 [38–46.5]	0.9
Extracellular Water (L) ^(2)^	45	22.5 [20.6–26.7]	22.7 [20.4–25]	0.97

n. number of patients. Variables reported as ^(1)^ mean (±standard deviation), ^(2)^ median and [interquartile range]. BMI, body mass index; BIA, bioelectrical impedance analysis.

**Table 3:** Sarcopenia diagnosis at T0 and T1 according to EWGSOP 2 criteria.

	n.	T0	T1	*p*-Value
Sarcopenia diagnosis ^(1)^	45			
- Sarcopenic		6 (13.3%)	6 (13.3%)	1
- Not sarcopenic		39 (86.7%)	39 (86.7%)	
Handgrip (kg) ^(2)^	45	25.2 (±8.8)	25.2 (±8.7)	0.95
ASM/h^2^ (kg/m^2^) ^(3)^	45	7.44 [6.46–8.23]	7.39 [6.47–8.15]	0.53
Gait Speed (m/s) ^(3)^	45	0.95 (±0.34)	0.96 (±0.34)	0.64

n. number of patients. ASM, appendicular skeletal muscle mass. Variables reported as ^(1)^ as absolute numbers and percentages n (%); ^(2)^ mean (±standard deviation); ^(3)^ median and [interquartile range].

**Table 4:** Blood and urine chemistry tests at T0 and T1.

	n.	T0	T1	*p*-Value
GFR (mL/min/1.73 m^2^) ^(1)^	45	16.8 (±4.9)	17 (±6.2)	0.66
Creatinine (umol/L) ^(2)^	45	291 [234–356]	296 [215–417]	0.57
Plasma urea (mmol/l) ^(1)^	45	22.5 (±7)	17.9 (±5.9)	**<0.001**
Uric acid (mmol/l) ^(2)^	44	0.34 [0.3–0.46]	0.38 [0.31–0.47]	0.27
Sodium (mmol/L) ^(1)^	45	139.3 (±2.7)	138.6 (±4.8)	0.33
Potassium (mmol/L) ^(2)^	45	4.4 (±0.5)	4.3 (±0.5)	0.14
Phosphorus (mmol/L) ^(2)^	44	1.3 [1.15–1.43]	1.27 [1.04–1.41]	0.09
Calcium (mmol/L) ^(2)^	44	2.35 [2.25–2.4]	2.33 [2.26–2.4]	0.12
PTH (ng/L)	35	119 (64–219)	99 (65–178)	0.64
Albumin (g/L) ^(1)^	45	38.9 (±3.9)	38.6 (±2.9)	0.73
Hemoglobin (g/L) ^(1)^	44	118.4 (±13.9)	116 (±11)	0.64
Blood glucose (mg/dl) ^(2)^	45	99 [92–117]	92 [86.5–118.3]	0.86
Proteinuria 24 h (g/24 h) ^(2)^	42	1.02 [0.53–2.8]	0.57 [0.29–2.14]	**0.01**

n. number of patients. Variables reported as ^(1)^ mean (±standard deviation), ^(2)^ median and [interquartile range].

**Table 5:** Food intakes at T0 and T1.

	n.	T0	T1	*p*-Value
Energy (kcal) ^(3)^	45	1700 [1523–1836]	1656 [1567–1815]	0.73
Proteins (g) ^(3)^	45	61.1 [56.5–70.1]	38.6 [32.5–42.3]	**<0.001**
Lipids (g) ^(3)^	45	64.1 [56.6–67.7]	66 [59.7–72.6]	**0.01**
Carbohydrates (g) ^(3)^	45	206.8 [180–228.5]	228.6 [202.3–250]	**<0.001**
Fiber (g) ^(3)^	45	17.4 [15.2–19.5]	18.6 [16.3–22.3]	**<0.001**
Sodium (mg) ^(3)^	45	965 [800–1223]	681 [575–822]	**<0.001**
Potassium (mg) ^(3)^	45	2409 [2061–2760]	2105 [1782–2412]	**<0.001**
Phosphorus (mg) ^(3)^	45	986 [855–1117]	666 [550–771]	**<0.001**
Use of free-protein food ^(1)^	45		42 (±91.1%)	
Prescribed diet	45			
- Energy (kcal) ^(2)^	45		1846 (±167.5)	
- Proteins (g) ^(2)^	45		38 (±5.7)	

n. number of patients. Variables reported as ^(1)^ as absolute number and percentages n.(%); ^(2)^ mean (±standard deviation); ^(3)^ median and [interquartile range].

## Data Availability

The data presented in this study are available on request from the corresponding author. The data are not publicly available due to privacy.
